# Operational definitions of asthma in recent epidemiological studies are inconsistent

**DOI:** 10.1186/2045-7022-4-24

**Published:** 2014-08-04

**Authors:** Ana Sá-Sousa, Tiago Jacinto, Luís Filipe Azevedo, Mário Morais-Almeida, Carlos Robalo-Cordeiro, António Bugalho-Almeida, Jean Bousquet, João Almeida Fonseca

**Affiliations:** 1 Center for research in health technologies and information systems.– CINTESIS, Universidade do Porto, Porto, Portugal; 2 Allergy Unit, Instituto CUF Porto e Hospital CUF Porto, Porto, Portugal; 3 Health Information and Decision Sciences Department – CIDES, Faculdade de Medicina, Universidade do Porto, Porto, Portugal; 4 Allergy and Clinical Immunology Department, Hospital CUF-Descobertas, Lisboa, Portugal; 5 Allergy and Clinical Immunology Department, Hospitais da Universidade de Coimbra, Coimbra, Portugal; 6 Clínica Universitária de Pneumologia-Faculdade de Medicina de Lisboa, Lisboa, Portugal; 7 Hôpital Arnaud de Villeneuve, Centre Hospitalier Universitaire Montpellier, Montpellier, France; 8 Centre de recherche en Epidémiologie et Santé des Populations, CESP Inserm U1018, Villejuif, France; 9 Faculdade de Medicina da Universidade do Porto, Rua Dr. Plácido da Costa, s/n, 4200-450 Porto, Portugal

**Keywords:** Asthma, Definition, Epidemiology, Prevalence, Review

## Abstract

**Objective:**

The best combination of questions to define asthma in epidemiological asthma studies is not known. We summarized the operational definitions of asthma used in prevalence studies and empirically assess how asthma prevalence estimates vary depending on the definition used.

**Methods:**

We searched the Thomson Reuters ISI Web of knowledge and included (1) cross-sectional studies (2) on asthma prevalence (3) conducted in the general population and (4) containing an explicit definition of asthma. The search was limited to the 100 most-cited papers or published since January 2010. For each paper, we recorded the asthma definition used and other variables. Then we applied the definitions to the data of the Portuguese National Asthma survey (INAsma) and of the 2005–2006 National Health and Nutrition Examination Survey (NHANES) computing asthma prevalence estimates for the different definitions.

**Results:**

Of 1738 papers retrieved, 117 were included for analysis. Lifetime asthma, diagnosed asthma and current asthma were defined in 8, 12 and 29 different ways, respectively. By applying definitions of current asthma on INAsma and NHANES data, the prevalence ranged between 5.3%-24.4% and 1.1%-17.2%, respectively.

**Conclusions:**

There is considerable heterogeneity in the definitions of asthma used in epidemiological studies leading to highly variable estimates of asthma prevalence. Studies to inform a standardized operational definition are needed. Meanwhile, we propose a set of questions to be reported when defining asthma in epidemiological studies.

## Introduction

The World Health Organization recommends assessing population needs concerning chronic respiratory diseases to better define adequate health policies [1]. Epidemiological studies at a population level, and prevalence studies in particular, are essential for this purpose.

The main multinational studies on asthma prevalence are the European Community Respiratory Health Survey (ECRHS) in adults [2], and the International Study of Asthma and Allergies in Childhood (ISAAC) in children and adolescents [3]. The European Union–funded Global Allergy and Asthma European Network (GA^2^LEN) recently conducted a large survey on the prevalence of airway and allergic diseases, built mainly on the questions and definitions used in the ECRHS [4].

Although the definitions used to diagnose asthma has a great impact on prevalence estimates [5], a standardized operational definition of asthma is still lacking. Asthma should ideally be defined by a combination of symptoms, clinical diagnosis, pulmonary function tests and studies of either bronchial hyperresponsiveness (BHR) or reversibility of airflow obstruction [6-8]. However, this is rarely feasible. In fact, in the context of population studies, only a few have combined the use of an initial symptoms questionnaire followed by clinical assessment including lung function tests in subsamples of symptomatic and asymptomatic individuals [2,3].

The aim of this paper is 1) to provide an overview of the operational definitions of asthma used in recent prevalence studies and 2) to empirically assess the impact of the definition used on the prevalence of asthma estimates using data from the first Portuguese National Asthma survey, *Inquérito Nacional sobre Asma* (INAsma) [9] and National Health and Nutrition Examination Survey (NHANES) 2005–2006 [10]. Based on this approach we propose a set of questions to be used when defining asthma in epidemiological studies, until a standardized operational definition based on prospective data can be established.

## Methods

### Literature search strategy and inclusion criteria

**Figure 1:**
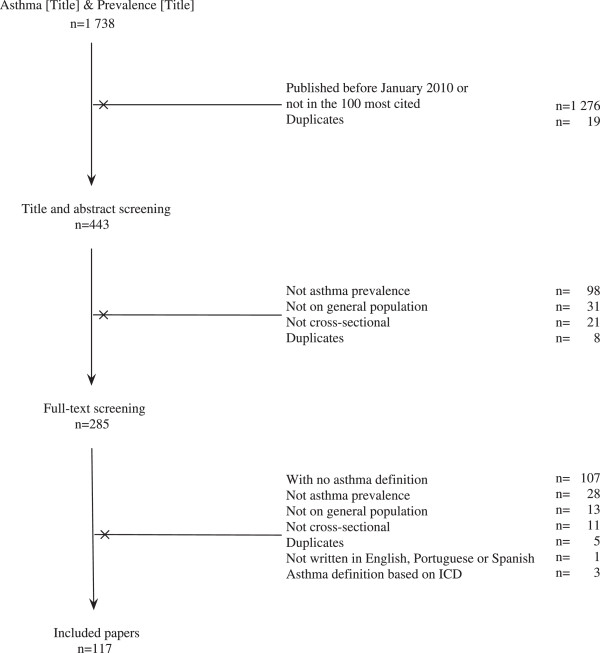
**Study selection.** From the 1 738 papers retrieved from the Web of Knowledge using the terms *asthma* and *prevalence* in the title; 117 were finally included in the study.

We performed a systematic literature search of the Thomson Reuters ISI Web of Knowledge. The initial search targeted papers with the terms “asthma” and “prevalence” in the title and the final selection included (i) cross-sectional studies (ii) on asthma prevalence (iii) conducted in the general population.To have a comprehensive overview of the definitions of asthma, with both the most recent definitions and most cited ones, we had two criteria in the papers selection: 1) The search was limited to the studies published between January 2010 and October 2013 and 2) the 100 most-cited papers on the topic were included in the initial search (Figure 1).

Papers written in languages other than English, Portuguese, Spanish or French were excluded, as were those without an explicit definition of asthma or with definitions of asthma based on the International Classification of Diseases (ICD).

#### Data extraction from published studies

Papers were screened by a single author (AS-S) in two phases (title and abstract and full-text screening) to exclude duplicates and articles that did not meet the eligibility criteria. Papers satisfying the inclusion criteria were examined and data on country, number and age of participants, site of screening, questionnaire used, prevalence value and 95% confidence interval (CI) and definition of asthma were recorded on a purpose-designed form. Doubts regarding the inclusion of papers and data extraction were clarified in agreement with a second investigator.

### Epidemiologic studies datasets

The *Inquérito Nacional sobre Asma* (INAsma) was conducted in 2010 under the auspices of the Portuguese Health Directorate and estimated a prevalence of current asthma (defined as affirmative to the question “Have you ever had asthma?” and at least one of 3 symptoms in the last 12 months: wheezing, waking with breathlessness or having an asthma attack) of 6.8% for the Portuguese population [9]. The INAsma survey comprised two phases: estimation of the prevalence of current asthma and estimation of the proportion of asthmatic patients with controlled disease. The INAsma survey and current study were reported in accordance with the STrengthening the Reporting of OBservational studies in Epidemiology (STROBE) statement guidelines [11].

The National Health and Nutrition Examination Survey (NHANES) 2005–2006 assessed the health and nutritional status of the United States population. And estimated a prevalence of asthma (defined as affirmative to the following questions: 1) Has a doctor or other health professional ever told you that you have asthma? 2) Do you still have asthma?) of 8.8% for the U.S. population [12].

Further details on databases can be found in Additional file 1.

### Data analysis

For each asthma definition recorded, we presented the corresponding prevalence value and 95% CI (if available) and computed the proportion of individuals with asthma using data from the INAsma survey and NHANES 2005–2006.

The definitions found were reproduced as they appeared in each paper and were grouped into 3 general subgroups: (i) current asthma (if the definition included current asthma features such as symptoms, current use of medication or the “still have asthma” expression); (ii) diagnosed asthma (if the definition included an asthma diagnosis by a health care professional or ever use of medication for asthma, but not any current asthma feature) and (iii) lifetime asthma (if the definition included having ever had asthma, but not any current asthma feature or asthma diagnosis).

**Figure 2:**
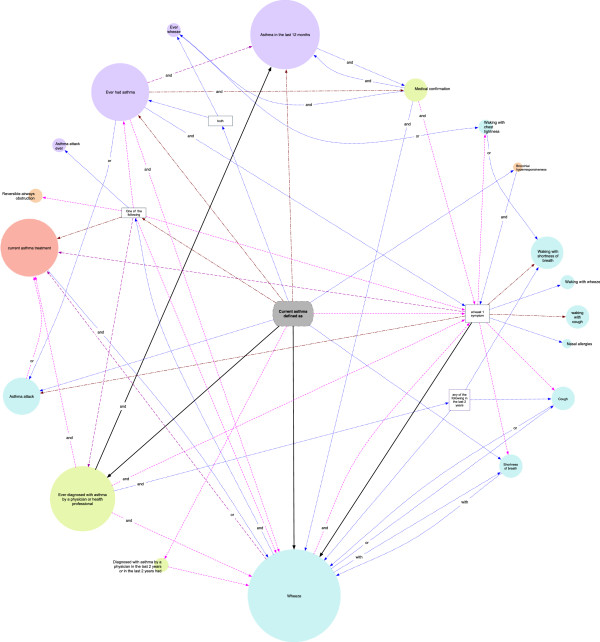
**Elements of the definitions of “Current asthma” connected in the order established by each studies.** Symptoms in the last 12 months are represented in blue, lung function tests in orange and physician diagnosis in green. The size of each element is proportional to the number of definitions using the element. The color of the lines indicates the number of times each connection was used: once (blue), twice (pink), three times (purple), four to eight times (red) and more than ten times (black). To simplify the diagram, *breathlessness* and *dyspnea* were considered the same.

Statistical analyses were performed using IBM SPSS Statistics v21 (2012 SPSS Inc., IBM Company, Chicago, US). The two phases of INAsma comprises data from 6 257 participants, both males and females from 0 to 98 years old. NHANES 2005–06 comprises data from 10 348 participants both males and females from 0 to 150 years old. Using the two phases of INAsma and NHANES 2005–2006 data, we estimated the proportion of individuals with 95% CI with asthma for each of the definitions in all applicable cases and when the necessary data were available.The elements used in each paper to define current asthma were plotted in a diagram (Figure 2). The size of the circles is proportional to the number of definitions in which the element is used. Different colors were attributed to definition elements depending if they were related to symptoms in the last 12 months, lung function tests or to physician diagnosis. Definitions elements were connected in the sequence established by the definition in each study. The number of times each connection was used is also indicated.

## Results

Of the 1738 papers retrieved from the Web of Knowledge database, 117 were included for analysis (Figure 1). The characteristics of the studies included are summarized in Additional file 2 and 3. They described prevalence studies conducted all over the world, on children (27.4%), on adolescents (10.3%), on children and adolescents (25.6%), on adults (25.6%), on elders (1.7%) and on all population(8.5%). Several questionnaires were applied, but the most common one was the ISAAC questionnaire, used in 45.5% of studies.

Lifetime asthma was defined in 34 papers, diagnosed asthma in 54 and current asthma in 61 (see Additional file 4). The 3 subgroups were defined in 8, 12 and 29 different ways, respectively (Tables 1 and 2).The definition of current asthma in each paper was based on a combination of elements. The most common elements were “wheeze in the last 12 months” (in 30 definitions) and “ever diagnosed with asthma by a physician or health professional” (in 21 definitions) (Figure 2). “Current asthma treatment” was used in 12 definitions. Other common symptoms were “shortness of breath at rest” (n = 8), “asthma attack” (n = 7), “shortness of breath/dyspnea” (n = 4), “nocturnal cough” (n = 4) and “cough” (n = 3) in the last 12 months. Only 3 papers used “bronchial hyperresponsiveness” or “reversibility of airflow obstruction” in their definition of asthma. The most common combination used to define current asthma was “Ever diagnosed with asthma by a health professional” together with “still have asthma” (n = 13).

**Table 1:** Definitions of “current asthma” and prevalence estimates computed using data from INAsma survey (n = 6257) and from NHANES 2009 (n = 10348)

**Current asthma definition**	**INAsma**	**NHANES 05/06**
	**% [95%CI]**	**% [95%CI]**
Ever had asthma *and* wheeze in the last 12 months [13]	9.3 [8.8-10.0]	n.a.
Ever had asthma *and* this was confirmed by a physician *and* at least one in the last 12 months: wheeze, waking with tightness in the chest, waking with breathlessness, waking with cough [14]	8.5 [7.7-9.3]*	n.a.
Ever had asthma *and* at least one of 3 symptoms in the last 12 months: wheezing, waking with breathlessness *or* having an asthma attack [9]	10.3 [9.5-11.0]	n.a.
Ever had asthma *and* still have asthma [15,16]	n.a.	n.a.
Ever had asthma *and* was this confirmed by a physician *and* wheeze *and/or* taking medication(s) for asthma or wheezing in the past 12 months [17]	n.a.	n.a.
At least one of the following: 1) Ever had asthma *and* was confirmed by a doctor *and* still have asthma *or* 2) significant bronchodilator response according to reversibility [18]	n.a.	n.a.
Ever had asthma *and* still have asthma *and* was confirmed by a physician [19]	n.a.	n.a.
At least one of the following: 1) Ever had asthma and wheezing in the last 12 months *or* 2) wheezing 4 times or more during the last 12 months *or* 3) physician diagnosis and wheezing in the last 12 months *or* 4) current use of inhalation steroids [20]	n.a.	n.a.
Diagnosed with asthma by a physician *and* wheeze in the last 12 months [21]	7.6 [6.9-8.3]*	1.7 [1.5-1.9]
Diagnosed with asthma by a physician a*nd* currently taking asthma medication [22]	7.4 [6.7-8.1]*	1.1 [0.9-1.3]
Diagnosed with asthma by a physician *and* use of asthma medication in the last 12 months *and* cough or wheeze in the last 12 months [23]	5.3 [4.7-5.9]^†^	n.a.
Diagnosed with asthma by a physician *and at least one:* currently taking asthma medication, attacks of shortness of breath *or* recurrent wheeze in the last 12 months [24]	6.3 [5.6-6.0]*^,‡^	n.a.
Diagnosed with asthma by a physician *and* still have asthma [25-37]	n.a.	8.4 [7.9-8.4]
Diagnosed with asthma by a physician *and* any of the following in the previous 2 years: wheezing following a common cold, dyspneal feeling at night or early in the morning, cough or wheeze during exercise [38]	n.a.	n.a.
Physician stated that (you) had asthma in the last 2 years *or* on 2 or more separate occasions had wheezing in the last 2 years [39,40]	n.a.	n.a.
At least one of the following: 1) Diagnosed with asthma by a physician a*nd* at least 1 symptom in the last 12 months: wheeze, wheezes with attacks of shortness of breath, nocturnal attacks of shortness of breath, use of asthma medication *or* 2) reversible airways obstruction [41]	n.a.	n.a.
Wheeze in the last 12 months [42-54]	22.9 [21.9-23.9]	12.9 [12.2-13.5]
Wheeze in the last 12 months *or* currently taking asthma medication [55]	24.4 [23.3-25.5]	12.9 [12.2-13.5]
Current wheeze *and* 1 of the following: 1) ever had an asthma attack *or* 2) ever had asthma *or* 3) taking medication(s) for asthma or wheeze [56]	9.3 [8.6-10.0]**	n.a.
Asthma attack *or* currently taking asthma medication [2]	9.0 [8.3-9.7]	n.a.
At least one in the last 12 months: Waking at night by shortness of breath, had an asthma attack *or* currently taking any medicines (including inhalers, aerosols or tablets) for asthma [57]	14.5 [13.6-15.4]	n.a.
At least one of the following symptoms: wheeze in the last 12 months, coughing constantly for more than 3 weeks in any time in your life, attack of asthma in the last 12 months, *or* nasal allergies including hay fever in the last 12 months [58]	n.a.	17.2 [16.5-17.9]^‡‡^
Wheeze in the last 12 months *and* at least 1 of the following in the last 12 months: any lifetime episode of asthma; wheezing during physical exercise; four or more episodes of wheezing in the previous year; *or* waking at night at least once a week due to wheezing [59]	n.a.	8.8 [8.3-9.3]^††^
Asthma in the last 12 months [60-66]	n.a.	n.a.
Had a condition which causes difficulty in breathing, with wheezing noises in the chest in the last 12 months [67]	n.a.	n.a.
At least one of the following: 1) any symptom in the last 12 months: wheezing, shortness of breath, coughing bouts *or* dyspnea *or* 2) previous physician-based diagnosis of asthma *or* 3) use of medication for asthma [68]	n.a.	n.a.
Both: 1) Ever whistling sound from chest *or* chest tightness *or* breathlessness in the morning, *and* 2) having suffered from asthma *or* having an attack of asthma in the past 12 months *or* using bronchodilators [69]	n.a.	n.a.
Bronchial hyperresponsiveness *and* asthma symptoms (wheeze, night cough, *or* shortness of breath at rest) within the previous 12 months [70]	n.a.	n.a.
At least one of the following: 1) Recent symptoms (wheeze or nocturnal cough) *or* 2) asthma medication use [71]	39.5 [38.2-40.7]	n.a.

*Questions related to this variable were only asked in the second phase of INAsma; therefore the total number of participants was corrected for non-response in phase II (total n = 4949). *n.a.* – not available – Questions related to this variable were not asked in the INAsma survey. ^†^“Cough” was interpreted as “waking with cough” for estimation purposes. ^‡^The variable was considered as “diagnosed with asthma by a physician and currently taking asthma medication and attacks of asthma or wheeze in the last 12 months” for estimation purposes. ^††^The variable was considered without the symptom “any lifetime episode of asthma” for estimation purposes. ^‡‡^The variable was considered without the symptom “attack of asthma in the last 12 months” for estimation purposes. **“ever had an asthma attack” was considered as “ever had an asthma attack in the last 12 months” for estimation purposes.

**Table 2:** Definitions of “Lifetime asthma” and “Diagnosed asthma” and prevalence estimates computed using data from INAsma survey (n = 6257) and from NHANES 2009 (n = 10348)

**Definition**	**INAsma**	**NHANES 05/06**
	**% [95%CI]**	**% [95%CI]**
**Lifetime asthma**		
Ever had asthma [3,9,14,16,17,24,51,54,66,72-89]	14.5 [13.6-15.4]	n.a.
Wheeze in the last 12 months *or* ever had asthma [90]	28.1 [27.0-29.2]	n.a.
Had a condition which causes difficulty in breathing, with wheezing noises in the chest [67]	n.a.	n.a.
Self-report of dyspnea and nocturnal dyspnea associated with wheezing, or attacks of dyspnea with wheezing, or physician-diagnosed asthma [91]	n.a.	n.a.
Ever had wheeze [92]	n.a.	n.a.
Report of simultaneous dyspnea and wheezing in the absence of upper respiratory infections [93]	9.5 [8.8-10.2]	n.a.
Ever had wheeze *and* cough more than other (children) *and* breathlessness at rest or exercise [94]	n.a.	n.a.
Ever had asthma *and/or* one of the following: wheeze, cough or acute shortness of breath due to external factors [95]	n.a.	n.a.
**Diagnosed asthma**		
Ever diagnosed with asthma by a physician or health professional [21,23,24,26,28,29,52,66,85-88,91,92,94,96-118]	9.3 [8.5-10.2]*	13.4 [12.7-14.1]
Diagnosed with asthma by a physician *or* wheezing in the last 12 months, apart from cold or the flu [119]	20.1 [19.0-21.2]*	24.5 [23.6-25.3]^†^
Diagnosed with asthma by a physician in the last 12 months [120,121]	n.a.	n.a.
Ever had asthma *and* medical confirmation [14,17,38,122]	9.3 [8.5-10.1]*	n.a.
Ever had asthma *and* use of medication for asthma [9]	8.2 [7.5-8.9]	n.a.
Ever diagnosed with asthma by a physician or health professional *and* Ever stayed over night in the hospital for wheezing [123]	2.5 [2.1-2.9]*	n.a.
Ever diagnosed with asthma by a physician or health professional *and* use of medication for asthma [47]	7.4 [6.7-8.1]*	1.1 [0.9-1.3]
History of asthma *and* physical examination *and* skin-prick tests *and* peak flow measurements [53]	n.a.	n.a.
History of asthma *and* physical signs of asthma (rhonchi or chest deformity) [124]	n.a.	n.a.
Diagnosed with asthma by a physician *or* symptoms (presence of wheezing, history of dry cough, recurrent wheeze, dyspnea/ recurrent difficulty in breathing or recurrent chest tightness) *with* clinical assessment [125]	n.a.	n.a.
Recurrent wheezing episodes, clinically confirmed by a physician, exactly a pediatrician or a pediatric pulmonologist [126]	n.a.	n.a.
Physician stated that (you) had asthma in the last 2 years, on 2 or more separate occasions had wheezing in the last 2 years, *or* had asthma prior to 2 years ago *or* on 2 or more separate occasions had wheezing prior to 2 years ago [39,40]	n.a.	n.a.

*Questions related to this variable were only asked in the second phase of INAsma; therefore the total number of participants was corrected for non-response in phase II (total n = 4949). *n.a.* – not available – Questions related to this variable were not asked in the INAsma survey. ^†^“apart from cold or the flu” was not considered for estimation purposes.

Most of the papers using “wheeze in the last 12 months” as an element to define current asthma described the prevalence of asthma in children, adolescents or both (n = 21,70% - data not shown).

The prevalence of asthma estimates based on the applicable definitions of current asthma ranged between 5.3% and 39.5% in the Portuguese population for the 13 (45%) applicable definitions and between 1.1% and 17.2% in U.S. population for the 7(24%) applicable definitions (Table 1).

**Figure 3:**
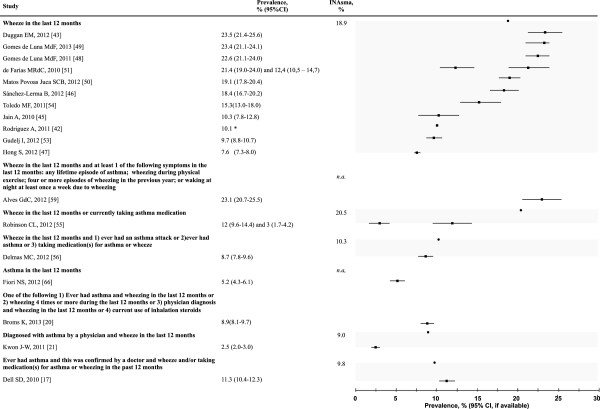
**Prevalence of asthma according to several definitions of the “Current asthma” based on ISAAC questionnaire.** Prevalence estimates for Portuguese children aged 6, 7, 13 and 14 years old were computed using data from the INAsma survey (also see Additional file 5). *95% CI not available. *n.a.* – not available. Proportion of people with asthma using the INAsma data are represented by •; prevalence stated in other studies are represented by ■. Lines represent the CI (when available). Shades gather the values for the same definition.

The definitions varied even in papers that used the same questionnaire. For instance, eight different definitions were used in 18 studies of the ISAAC questionnaire (see Additional file 5 and Figure 3). Using the eight variants of the definition of current asthma based on the ISAAC questionnaire, the proportion of children and adolescents with asthma estimated from the INAsma data varied between 9.0% and 20.5% (Figure 3).

Concerning definitions of lifetime asthma, three definitions could be used with data from INAsma with prevalence estimates ranging between 9.5% and 28.1%; none of the definitions were applicable to NHANES data (Table 2).

## Discussion

The operational definitions of asthma varied extensively among studies and do not allow to compare estimates between studies, even if similar data collection methods were used. Additionally, when we applied the definitions of current asthma to two datasets from population studies, we found that the prevalence of asthma ranged from 5.3% to 39.5% in Portuguese population and from 1.1% to 17.2% in U.S. population depending on the definition used. Current asthma was most frequently defined by the combination of “ever diagnosed with asthma by a health professional” with “still having asthma”. Wheeze was the most frequently used current symptom, especially in children.

The Web of Knowledge was the only database used for the search. While this may be seen as a limitation of the present study, this approach allowed the inclusion of the most-cited papers on asthma prevalence conducted in both children and adults.

The wide variability of the estimates computed for different definitions is in accordance with the results of a study in children [5]. The variety of definitions identified in the present study illustrates both the difficulty of identifying people with asthma by questionnaire and the difficulty of establishing an internationally accepted definition, even when the same questionnaire is used.

The few published studies that have validated asthma questionnaires, used BHR tests or a physician’s diagnosis as gold-standard [8,127,128]. These reports concluded that questions on wheeze are the most sensitive and questions such as *“*Have you ever had asthma*?”* or questions on “waking with attacks of shortness of breath*”* and “morning tightness*”* have high specificity for asthma [8,127,128]. In our review, wheeze was the most commonly used symptom, especially in children. However, it should be noted that a diagnosis of asthma based on wheeze is only accurate during some stages of childhood [129,130] and the use of a comprehensive description of asthma symptoms might reduce the risk of misclassification of asthma in children. Pekkanen and coworkers also suggested that definitions using a combination of asthma symptoms increase specificity against self-report of ever having asthma and BHR and that the use of a continuous asthma score based on asthma symptoms increases both the specificity and power for predicting asthma [7].

The present study shows that prior asthma diagnosis by a health professional was commonly used to define asthma. However, in some countries and cultural contexts, the use of prior diagnosis may lead to an under- or overestimation of asthma prevalence [131]. Attention to this potential problem is especially important in multinational studies and, in fact, ISAAC [3] and ECRHS [2] do not consider a prior diagnosis definitive for asthma.

**Figure 4:**
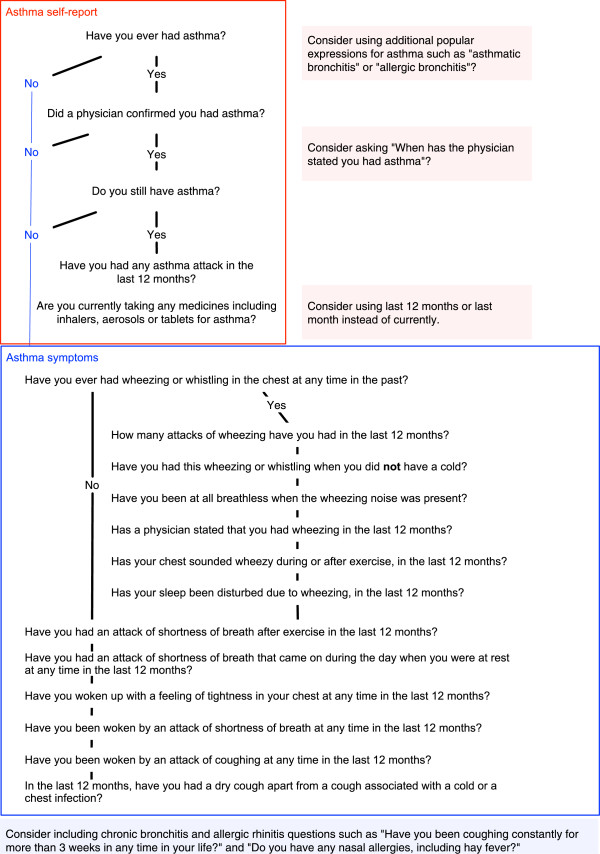
Questions to be asked and independently reported.

Standardized definitions are necessary for prevalence comparisons worldwide. This should be achieved by the validation of self-reported features, against objective measure, such as (i) variability of airflow obstruction, reversibility and BHR, (ii) physician classification including current therapy and (iii) persistence of diagnosis at clinical long-term follow-up. By reporting the sensitivity and specificity of different approaches, these validation studies would inform the decision on a standardized operational definition of asthma for epidemiological purposes.Based on the data presented, we propose an initial comprehensive set of questions (Figure 4) that can be used in validation studies while allowing comparisons with previous prevalence studies of asthma. As this is an early step to achieve a standardized definition of asthma, we suggest that at this time, the data items proposed in Figure 4 should be reported separately to increase the inter-study comparability.

## Conclusions

The operational definition of asthma has an important effect on estimates of asthma prevalence in epidemiologic studies. There is a need for an accurate standardized operational definition of asthma. To inform this definition it is important to estimate the sensibility and specificity of a set of questions (Figure 4) against reversibility/variability of the airflow obstruction, BHR and clinical assessment.

## Abbreviations

BHR: Bronchial hyperresponsiveness; CI: Confidence Interval; ECRHS: European Community Respiratory Health Survey; GA^2^LEN: Global Allergy and Asthma European Network; ICD-9: International Classification of Diseases; INAsma: Inquérito Nacional sobre Asma (Portugal); ISAAC: International Study of Asthma and Allergies in Childhood; NHANES: National Health and Nutrition Examination Survey (USA).

## Competing interests

The authors declare that they have no competing interests.

## Authors’ contributions

AS-S participated in data collection, analysis and wrote the manuscript draft, LFA participated in study design and reviewed the manuscript, TJ, CRC, MMA and ABA reviewed the manuscript and JB provided critical review during the project and reviewed the manuscript, JAF is responsible for the INAsma project and participated in all stages and tasks of the present study. All authors have read and approved the final manuscript.

## Supplementary Material

Additional file 1 Databases details. Click here for file

Additional file 2 Summary of the included studies. Click here for file

Additional file 3 Definitions in the ten most cited and the ten most recent papers from the 117 included by Average citations per year at the end of 2013. Click here for file

Additional file 4 **Studies presenting three different subgroups of the definition of asthma.** Of the 117 papers included, 34 provided a definition for lifetime asthma, 54 for diagnosed asthma and 61 for current asthma; 5 papers defined the three types of asthma. Click here for file

Additional file 5 Summary of studies with Current asthma definitions that used the International Study of Asthma and Allergies in Childhood (ISAAC) questionnaire (n = 18). Click here for file
