# Supplementary material for: Operational definitions of asthma in recent epidemiological studies are inconsistent
Source: Clin Transl Allergy. 2014 Aug 4;4:24. doi: 10.1186/2045-7022-4-24 (PMC4136946; doi:10.1186/2045-7022-4-24)
Supplement: Additional file 1 — Databases details. [file 2045-7022-4-24-S1.pdf]

INAsma database

The *Inquérito Nacional sobre Asma* (INAsma), conducted in 2010 was the first nationwide study on asthma that uses standardized methods. The survey comprises two phases. In the first phase, aiming to evaluate asthma prevalence we estimated that 7% of the Portuguese population had current asthma [1] ; in the second phase we estimated that half of the Portuguese asthma patients had their disease controlled (paper under review).

This was a cross-sectional, population-based, telephone interview survey including all municipalities of Portugal. To obtain a representative sample of the population, a stratified cluster sampling design was used as previously described [1]. Data were obtained for 6 257 respondents.

Interview components

The INAsma interviews include demographic, socioeconomic, and allergic diseases symptoms (based on the Portuguese version of the GA2LEN survey), asthma and allergic rhinitis control (based on CARAT), healthcare utilization, medication use, absenteeism, quality of life, physical activity and smoking habits.

Data Collection Methodology

A private company administered the questionnaire through Computer Assisted Telephone Interviews (CATI) performed by trained and experienced interviewers. Interviews were conducted between March and October of 2010, mostly between 17:00–22:00 h in weekdays and 11:00–22:00 h in weekends and holidays. Each telephone number was not abandoned before a minimum of ten attempts in different occasions.

Interviewer Training, quality control monitoring and data consistency check

Interviewers were selected based on their previous experience on health-related data collection; each question was discussed in training sessions held between researchers and all interviewers; a research assistant was present in

the setup, training and daily work of the interviewers, motivating and checking the compliance with the standardized operational procedures.

Data validity was periodically verified soon after being collected and custom statistic algorithms were used to detect extreme, illogical and missing values.

The clarity of the questionnaire and its telephonic administration was assessed in a pilot study with 25 individuals before starting the data collection.

NHANES 2005-2006 database

The National Health and Nutrition Examination Survey (NHANES) is a program of studies designed to assess the health and nutritional status of adults and children in the United States. The survey is unique in that it combines interviews and physical examinations.

The NHANES program began in the early 1960s and has been conducted as a series of surveys focusing on different population groups or health topics. In 1999, the survey became a continuous program that has a changing focus on a variety of health and nutrition measurements to meet emerging needs. The survey examines a nationally representative sample of about 5,000 persons each year. These persons are located in counties across the country, 15 of which are visited each year.

Further details on survey design can be found in http://www.cdc.gov/nchs/nhanes/about_nhanes.htm.

Interview components

The NHANES interview includes demographic, socioeconomic, dietary, and health-related questions. The examination component consists of medical, dental, and physiological measurements, as well as laboratory tests administered by highly trained medical personnel.

Data Collection Methodology

Household Interview and Mobile Examination Center (MEC) Interview data were recorded electronically using computerized questionnaire forms that

were programmed using Blaise© software. The Household Interview questionnaires were programmed in Blaise© and installed on pentop computers for use in the field. The MEC interviews were also administered in an automated format, but the interview module formats included non-Blaise© software. After data collection was completed, the interview data files were transmitted electronically to a central survey database system.

Interviewer Training, quality control monitoring and data consistency check

All interviewers completed a comprehensive two-week training program. Many of the interviewers had prior interviewing experience. NHANES interviewer training included role-playing exercises and practice interviews, all of which were monitored by NCHS and contractor staff. Detailed information about the interviewer training requirements is found in the NHANES Household Interviewer Training Manual.

Extensive pretesting was completed prior to implementing the questionnaires in the field. A full "dress rehearsal" pilot test was conducted with the field and examination staff, to test all of the systems and procedures. Several types of quality control monitoring methods were implemented and interviewers were frequently accompanied on interviews and observed to verify that the interview protocol was administered correctly. Interviewers were retrained on survey procedures if necessary.

The NHANES CAPI systems had built-in edit and range checks for most questions that have open-ended response options. When unusual or unrealistic responses were recorded, the interviewer was alerted immediately and instructed to verify or edit the initial response.

1. Sa-Sousa A, Morais-Almeida M, Azevedo LF, Carvalho R, Jacinto T, Todo-Bom A, et al. Prevalence of asthma in Portugal - The Portuguese National Asthma Survey. Clin Transl Allergy. 2012; **2**: 15.
