# Supplementary material for: Operational definitions of asthma in recent epidemiological studies are inconsistent
Source: Clin Transl Allergy. 2014 Aug 4;4:24. doi: 10.1186/2045-7022-4-24 (PMC4136946; doi:10.1186/2045-7022-4-24)
Supplement: Additional file 2 — Summary of the included studies. [file 2045-7022-4-24-S2.pdf]

Additional File 2 - Summary of the included studies

Table 1: Summary of the included studies (n=117) by definition of asthma

	**Lifetime asthma** [1-34]	**Diagnosed asthma** [2, 4, 17, 21-26, 30, 31, 33, 35-76]	**Current asthma** [2-4, 7, 9, 17, 21, 22, 29, 40, 41, 47, 54, 56, 63, 67, 70, 71, 75-117]
**Papers, n**	34	54	61
**Operational definitions, n**	8	12	29
**Prevalence of asthma, %**	1.4-28.2	1.0-38.6	1.5-29.4
**Questionnaires used**	ATS<br>ECRHS<br>FinEsS<br>ISAAC<br>MRC<br>NHIS<br>OLIN	ATS<br>ATS-DLD<br>BOLD<br>BPAS<br>CRQ<br>ECRHS<br>FinEsS<br>GEIRD<br>ISAAC<br>MRC<br>National asthma prevalence study (Bangladesh)<br>PNAD<br>National Maternal and Infant Health Survey<br>NHANES<br>OLIN<br>WHS	ATS<br>ATS-DLD<br>BOLD<br>ECRHS<br>ISAAC<br>IUATLD<br>MRC<br>National asthma prevalence study (Bangladesh)<br>NCHS<br>NFHS<br>NHANES<br>NHIS<br>NYC-NAAS<br>PNAD
**Country/Region**	Austria<br>Australia<br>Brazil<br>Canada<br>China<br>Cyprus<br>Denmark<br>Greece<br>Italy<br>Japan<br>Norway<br>Portugal<br>Scandinavia and Eastern Europe<br>South Korea<br>Spain<br>Sweden<br>United Arab Emirates<br>United Kingdom<br>United States<br>Vietnam	Australia<br>Bangladesh<br>Brazil<br>Canada<br>China<br>Colombia<br>Croatia<br>Finland<br>Germany<br>Greece<br>Iran<br>Japan<br>New Zealand<br>Portugal<br>Saudi Arabia<br>Serbia and Montenegro<br>Singapore<br>South Korea Sweden<br>Thailand<br>United Kingdom<br>United States<br>Ukraine<br>Vietnam	Australia<br>Bangladesh<br>Brazil<br>Canada<br>Croatia<br>Ecuador<br>European Community<br>Greece<br>India<br>Ireland<br>Japan<br>Peru<br>Portugal<br>Rwanda<br>South Korea<br>Spain<br>Sweden<br>Turkey<br>United Arab Emirates<br>United Kingdom<br>United States
**Data collection / questionnaire administration method**	Telephone<br>Post<br>School<br>Door-to-door	Telephone<br>Post<br>School<br>Primary care unit<br>Door-to-door<br>Mobile unit	Telephone<br>Post<br>School<br>Primary care unit<br>Door-to-door<br>Mobile unit<br>Public place

The asthma definitions were grouped into 3 general subgroups: lifetime asthma (if the respondent reported ever having had asthma), diagnosed asthma (if the respondent reported asthma diagnosed by a health professional) and current asthma (if the respondent reported having had asthma features in the 12 months prior to the interview). ATS, American Thoracic Society; ATS-DLD, American Thoracic Society-Division of Lung Disease; BOLD, Burden of Lung Disease; BPAS, Brief Pediatric Asthma Screen; CSGA, Collaborative Studies on the Genetics of Asthma; CRQ, Children's Respiratory Questionnaire; ECRHS, European Community Respiratory Health Survey; FinEsS, Finnish, Estonian and Swedish respiratory Survey; ISAAC, International Study of Asthma and Allergies in Childhood; IUATLD, International Union Against Tuberculosis and Lung Disease; MRC, Medical Research Council respiratory questionnaire; NCHS, Five data sources from the National Center for Health Statistics; NFHS, National Family Health Survey; NHANES, National Health and Nutrition Examination Survey; NHIS, National Health Interview Survey; NYC-NAAS, New York City – Neighborhood Asthma and Allergy Study; PNAD, National Household Sample Survey-Pesquisa Nacional por Amostra de Domicílios; OLIN, Obstructive Lung Disease In Northern Sweden; WHS, World Health Survey.

Table 2: The 117 included papers by Average citation per year by the end of 2013.

First Author	Year	Journal	Times cited	Average citations per year
Pearce, N.	2007	Thorax	291	41.57
Asher, M. I.	1998	European Respiratory Journal	644	40.25
Akinbami, L. J.	2002	Pediatrics	408	34
Burney, P.	1996	European Respiratory Journal	514	28.56
Burr, M. L.	1989	Archives Of Disease In Childhood	644	25.76
Burney, P. G. J.	1990	British Medical Journal	482	20.08
Moorman, J. E.	2011	Morbidity And Mortality Weekly Report. Surveillance Summaries	59	19.67
Vonmutius, E.	1992	British Medical Journal	331	15.05
Gergen, P. J.	1988	Pediatrics	383	14.73
Robertson, C. F.	1991	British Medical Journal	313	13.61
Weitzman, M.	1992	JAMA-Journal Of The American Medical Association	280	12.73
Upton, M. N.	2000	British Medical Journal	178	12.71
Crain, E. F.	1994	Pediatrics	247	12.35
Mortz, C. G.	2001	British Journal Of Dermatology	154	11.85
Kaur, B.	1998	British Medical Journal	177	11.06
To, T.	2012	BMC Public Health	22	11
Galassi, C.	2006	Pediatrics	85	10.63
Litonjua, A. A.	1999	Pediatric Pulmonology	148	9.87
Downs, S. H.	2001	Archives Of Disease In Childhood	126	9.69
Anderson, H. R.	1994	British Medical Journal	187	9.35
Haby, M. M.	2001	Thorax	121	9.31
Woolcock, A. J.	1987	Thorax	243	9
Zollner, I. K.	2005	Thorax	81	9
Arif, A. A.	2003	European Respiratory Journal	93	8.45
Akinbami, L. J.	2011	National Health Statistics Reports	25	8.33
Rodriguez, M. A.	2002	Archives Of Pediatrics & Adolescent Medicine	99	8.25
Pearce, N.	2000	European Respiratory Journal	112	8
Strachan, D. P.	1994	Archives Of Disease In Childhood	155	7.75
Bjorksten, B.	1998	European Respiratory Journal	120	7.5
Miller, J. E.	2000	American Journal Of Public Health	105	7.5
Leung, R.	1997	European Respiratory Journal	118	6.94
Guo, Y. L. L.	1999	Environmental Health Perspectives	103	6.87
Goh, D. Y. T.	1996	Archives Of Disease In Childhood	118	6.56
Peat, J. K.	1992	British Medical Journal	141	6.41
Joseph, C. L. M.	1996	Journal Of Pediatrics	105	5.83
Oraka, E.	2010	Chest	23	5.75
Kwon, J.-W.	2011	Allergy Asthma & Immunology Research	17	5.67
Bjerg, A.	2010	Allergy	22	5.5
Krstic, G.	2011	Plos One	16	5.33
Akinbami, L. J.	2010	Environmental Research	21	5.25
Clifford, R. D.	1989	Archives Of Disease In Childhood	125	5
Waite, D. A.	1980	Clinical Allergy	165	4.85
Studnicka, M.	1997	European Respiratory Journal	81	4.76
Ma, J.	2010	Allergy	19	4.75
James, A. L.	2010	European Respiratory Journal	19	4.75
Mak, H.	1982	Journal Of Allergy And Clinical Immunology	146	4.56
Wennergren, G.	2010	Pediatric Allergy And Immunology	16	4
Sanchez-Lerma, B.	2012	Journal Of Investigational Allergology And Clinical Immunology	8	4
Arnedo-Pena, A.	2011	International Journal Of Biometeorology	11	3.67
Duggan, E. M.	2012	Pediatric Allergy And Immunology	7	3.5
Barry, A. C.	2010	Journal Of Asthma	11	2.75
Fukutomi, Y.	2010	International Archives Of Allergy And Immunology	10	2.5
Rodriguez, A.	2011	Thorax	7	2.33
Ekerljung, L.	2010	International Journal Of Tuberculosis And Lung Disease	9	2.25
Bai, J.	2010	Biomedical And Environmental Sciences	9	2.25
Hansen, T. E.	2013	Acta Paediatrica	2	2
Dell, S. D.	2010	Canadian Respiratory Journal	8	2
Lukrafka, J. L.	2010	Journal Of Asthma	8	2
Musafiri, S.	2011	Respiratory Medicine	6	2

First Author	Year	Journal	Times cited	Average citations per year
Chittleborough, C. R.	2010	Respirology	6	1.5
Sa-Sousa, A.	2012	Clinical And Translational Allergy	3	1.5
Fukutomi, Y.	2011	Allergology International	4	1.33
Julia-Serda, G.	2011	International Journal Of Tuberculosis And Lung Disease	4	1.33
Sousa, S. I. V.	2011	Environment International	4	1.33
Andrea Rodriguez, L.	2010	Biomedica	5	1.25
Wehrmeister, F. C.	2010	Cadernos De Saude Publica	5	1.25
Frazier, J. C.	2012	Preventing Chronic Disease	2	1
de Marco, R.	2013	Plos One	1	1
Agrawal, S.	2012	Journal Of Asthma	2	1
Koshy, G.	2010	Journal Of Public Health	4	1
Lam, H. T.	2011	Respiratory Medicine	3	1
Pegas, P. N.	2011	Revista Portuguesa De Pneumologia	3	1
Yeh, K.-W.	2011	Asian Pacific Journal Of Allergy And Immunology	3	1
Alves, G. d. C.	2012	Cadernos De Saude Publica	2	1
Ekici, A.	2012	Journal Of Asthma	2	1
Fedortsiv, O.	2012	Advances In Medical Sciences	2	1
Robinson, C. L.	2012	Bmj Open	2	1
Kim, S. Y.	2013	Lung	1	1
Alsowaidi, S.	2010	Respiration	4	1
Ekerljung, L.	2010	Clinical Respiratory Journal	4	1
Delmas, M. C.	2012	Revue Des Maladies Respiratoires	2	1
Akinbami, L. J.	2012	NCHS Data Brief	2	1
Kainu, A.	2013	Primary Care Respiratory Journal	1	1
Jain, A.	2010	Indian Journal Of Pediatrics	3	0.75
Anthracopoulos, M. B.	2011	Allergy And Asthma Proceedings	2	0.67
Anthracopoulos, M. B.	2011	Acta Paediatrica	2	0.67
Bryant-Stephens, T.	2012	Journal Of Asthma	1	0.5
Oraka, E.	2012	Journal Of Asthma	1	0.5
Al Ghobain, M. O.	2012	Bmc Public Health	1	0.5
Gudelj, I.	2012	Medical Science Monitor	1	0.5
de Farias, M. R. d. C.	2010	Brazilian Journal Of Epidemiology	2	0.5
Parasuramalu, B. G.	2010	Indian Journal Of Public Health	2	0.5
Coker, T. R.	2012	Health Services Research	1	0.5
Fiori, N. S.	2012	Cadernos De Saude Publica	1	0.5
Matos Povoas Juca, S. C. B.	2012	Cadernos De Saude Publica	1	0.5
Kolokotroni, O.	2011	BMC Public Health	1	0.33
de Luna, M. d. F. G.	2011	Cadernos De Saude Publica	1	0.33
de Sousa, J. C.	2011	BMC Public Health	1	0.33
Toledo, M. F.	2011	Allergologia Et Immunopathologia	1	0.33
Zivkovic, Z.	2010	World Journal Of Pediatrics	1	0.25
Shakurnia, A. H.	2010	Eastern Mediterranean Health Journal	1	0.25
Agrawal, S.	2013	International Journal Of Tuberculosis And Lung Disease	0	0
Broms, K.	2013	BMC Public Health	0	0
Bennoor, K. S.	2011	Respirology	0	0
Cornell, A. G.	2011	American Journal Of Respiratory And Critical Care Medicine	0	0
de Luna, M. d. F. G.	2013	Jornal Brasileiro De Pneumologia	0	0
de Sousa, C. A.	2012	Revista De Saude Publica	0	0
Hong, S.	2012	Environmental Health And Toxicology	0	0
Hwang, G.-S.	2012	Asia-Pacific Journal Of Public Health	0	0
Jie, Y.	2013	Polish Journal Of Environmental Studies	0	0
Lopez-Silvarrey-Varela, A.	2011	Archivos De Bronconeumologia	0	0
Mahboub, B. H.	2012	Bmc Pulmonary Medicine	0	0
Moreira, S. M.	2010	European Journal Of Medical Research	0	0
Nriagu, J.	2012	Science Of The Total Environment	0	0
Rerksuppaphol, S.	2012	Pediatric Reports	0	0
Zahran, H. S.	2011	JAMA-Journal Of The American Medical Association	0	0
Zobeiri, M.	2011	Acta Medica Iranica	0	0

References

1. Studnicka M, Hackl E, Pischinger J, Fangmeyer C, Haschke N, Kuhr J, Urbanek R, Neumann M, Frischer T: **Traffic-related NO2 and the prevalence of asthma and respiratory symptoms in seven year olds.** *Eur Respir J* 1997, **10:**2275-2278.
2. Sa-Sousa A, Morais-Almeida M, Azevedo LF, Carvalho R, Jacinto T, Todo-Bom A, Loureiro C, Bugalho-Almeida A, Bousquet J, Fonseca JA: **Prevalence of asthma in Portugal - The Portuguese National Asthma Survey.** *Clin Transl Allergy* 2012, **2:**15-15.
3. de Farias MRdC, Rosa AM, Hacon SdS, de Castro HA, Ignotti E: **Prevalence of asthma in schoolchildren in Alta Floresta- a municipality in the southeast of the Brazilian Amazon.** *Rev Bras Epidemiol* 2010, **13:**49-57.
4. Fiori NS, Goncalves H, Dumith SC, Dropa Chrestani Cesar MA, Menezes AMB, Cardozo Macedo SE: **Ten-year trends in prevalence of asthma in adults in southern Brazil: comparison of two population-based studies.** *Cad Saude Publica* 2012, **28:**135-144.
5. Mortz CG, Lauritsen JM, Bindslev-Jensen C, Andersen KE: **Prevalence of atopic dermatitis, asthma, allergic rhinitis, and hand and contact dermatitis in adolescents. The Odense Adolescence Cohort Study on Atopic Diseases and Dermatitis.** *Br J Dermatol* 2001, **144:**523-532.
6. Kolokotroni O, Middleton N, Nicolaou N, Pipis S, Priftis KN, Milton DK, Yiallouros PK: **Temporal changes in the prevalence of childhood asthma and allergies in urban and rural areas of Cyprus: results from two cross sectional studies.** *Bmc Public Health* 2011, **11**.
7. Toledo MF, Rozov T, Leone C: **Prevalence of asthma and allergies in 13-to 14-year-old adolescents and the frequency of risk factors in carriers of current asthma in Taubate, Sao Paulo, Brazil.** *Allergol Immunopathol (Madr)* 2011, **39:**284-290.
8. Pearce N, Aiet-Khaled N, Beasley R, Mallol J, Keil U, Mitchell E, Robertson C, Grp IPTS: **Worldwide trends in the prevalence of asthma symptoms: phase III of the International Study of Asthma and Allergies in Childhood (ISAAC).** *Thorax* 2007, **62:**758-766.
9. Crain EF, Weiss KB, Bijur PE, Hersh M, Westbrook L, Stein REK: **An estimate of the prevalence of asthma and wheezing among inner-city children.** *Pediatrics* 1994, **94:**356-362.
10. Upton MN, McConnachie A, McSharry C, Hart CL, Smith GD, Gillis CR, Watt GCM: **Intergenerational 20 year trends in the prevalence of asthma and hay fever in adults: the Midspan family study surveys of parents and offspring.** *Br Med J* 2000, **321:**88-92.
11. Asher MI, Anderson HR, Stewart AW, Crane J, Ait-Khaled N, Anabwani G, Anderson HR, Beasley R, Bjorksten B, Burr ML, et al: **Worldwide variations in the prevalence of asthma symptoms: the International Study of Asthma and Allergies in Childhood (ISAAC).** *Eur Respir J* 1998, **12:**315-335.
12. Bjorksten B, Dumitrascu D, Foucard T, Khetsuriani N, Khaitov R, Leja M, Lis G, Pekkanen J, Priftanji A, Riikjarv MA: **Prevalence of**

childhood asthma, rhinitis and eczema in Scandinavia and Eastern Europe. *Eur Respir J* 1998, **12:**432-437.
13. Galassi C, De Sario M, Biggeri A, Bisanti L, Chellini E, Ciccone G, Petronio MG, Piffer S, Sestini P, Rusconi F, et al: **Changes in prevalence of asthma and allergies among children and adolescents in Italy: 1994-2002.** *Pediatrics* 2006, **117:**34-42.
14. Leung R, Wong G, Lau J, Ho A, Chan JKW, Choy D, Douglass C, Lai CKW: **Prevalence of asthma and allergy in Hong Kong schoolchildren: An ISAAC study.** *Eur Respir J* 1997, **10:**354-360.
15. Robertson CF, Heycock E, Bishop J, Nolan T, Olinsky A, Phelan PD: **PREVALENCE OF ASTHMA IN MELBOURNE SCHOOLCHILDREN - CHANGES OVER 26 YEARS.** *Br Med J* 1991, **302:**1116-1118.
16. Pegas PN, Alves CA, Scotto MG, Evtyugina MG, Pio CA, Freitas MC: **Risk factors and prevalence of asthma and rhinitis among primary school children in Lisbon.** *Rev Port Pneumol* 2011, **17:**109-116.
17. Dell SD, Foty RG, Gilbert NL, Jerret M, To T, Walter SD, Stieb DM: **Asthma and allergic disease prevalence in a diverse sample of Toronto school children: results from the Toronto Child Health Evaluation Questionnaire (T-CHEQ) Study.** *Can Respir J* 2010, **17:**e1-6.
18. Lopez-Silvarrey-Varela A, Pertega-Diaz S, Rueda-Esteban S, Manuel Sanchez-Lastres J, Angel San-Jose-Gonzalez M, Sampedro-Campos M, Perez-Castro T, Garnelo-Suarez L, Bamonde-Rodriguez L, Lopez-Silvarrey-Varela J, Gonzalez-Barcala J: **Prevalence and Geographic Variations in Asthma Symptoms in Children and Adolescents in Galicia (Spain).** *Arch Bronconeumol* 2011, **47:**274-282.
19. Pearce N, Sunyer J, Cheng S, Chinn S, Bjorksten B, Burr M, Keil U, Anderson HR, Burney P, Com ISCE: **Comparison of asthma prevalence in the ISAAC and the ECRHS.** *Eur Respir J* 2000, **16:**420-426.
20. Kaur B, Anderson HR, Austin J, Burr M, Harkins LS, Strachan DP, Warner JO: **Prevalence of asthma symptoms, diagnosis, and treatment in 12-14 year old children across Great Britain (international study of asthma and allergies in childhood, ISAAC UK).** *Br Med J* 1998, **316:**118-124.
21. Fukutomi Y, Nakamura H, Kobayashi F, Taniguchi M, Konno S, Nishimura M, Kawagishi Y, Watanabe J, Komase Y, Akamatsu Y, et al: **Nationwide Cross-Sectional Population-Based Study on the Prevalences of Asthma and Asthma Symptoms among Japanese Adults.** *Int Arch Allergy Immunol* 2010, **153:**280-287.
22. Ekerljung L, Andersson A, Sundblad BM, Ronmark E, Larsson K, Ahlstedt S, Dahlen SE, Lundback B: **Has the increase in the prevalence of asthma and respiratory symptoms reached a plateau in Stockholm, Sweden?** *Int J Tuberc Lung Dis* 2010, **14:**764-771.
23. Ekerljung L, Sundblad B-M, Ronmark E, Larsson K, Lundback B: **Incidence and prevalence of adult asthma is associated with low socio-economic status.** *Clin Respir J* 2010, **4:**147-156.

24. Lam HT, Ronmark E, Tu'o'ng NV, Ekerljung L, Chuc NTK, Lundback B: **Increase in asthma and a high prevalence of bronchitis: results from a population study among adults in urban and rural Vietnam.** *Respir Med* 2011, **105:**177-185.
25. James AL, Knuiman MW, Divitini ML, Hui J, Hunter M, Palmer LJ, Maier G, Musk AW: **Changes in the prevalence of asthma in adults since 1966: the Busseltion health study.** *Eur Respir J* 2010, **35:**273-278.
26. Wennergren G, Ekerljung L, Alm B, Eriksson J, Lotvall J, Lundback B: **Asthma in late adolescence - farm childhood is protective and the prevalence increase has levelled off.** *Pediatr Allergy Immunol* 2010, **21:**806-813.
27. Arnedo-Pena A, Garcia-Marcos L, Fuertes Fernandez-Espinar J, Bercedo-Sanz A, Aguinaga-Ontoso I, Gonzalez-Diaz C, Carvajal-Uruena I, Busquet-Monge R, Morales Suarez-Varela M, Garcia de Andoin N, et al: **Sunny hours and variations in the prevalence of asthma in schoolchildren according to the International Study of Asthma and Allergies (ISAAC) Phase III in Spain.** *Int J Biometeorol* 2011, **55:**423-434.
28. Alsowaidi S, Abdulle A, Bernsen R: **Prevalence and Risk Factors of Asthma among Adolescents and Their Parents in Al-Ain (United Arab Emirates).** *Respiration* 2010, **79:**105-111.
29. Mak H, Johnston P, Abbey H, Talamo RC: **Prevalence of athma and health-service utilization of asthmatic-children in an inner-city** *J Allergy Clin Immunol* 1982, **70:**367-372.
30. Hwang G-S, Choi J-W, Yoo Y, Choung J-T, Yoon C-S: **Residential Environmental Risk Factors for Childhood Asthma Prevalence in Metropolitan and Semirural Cities in Korea.** *Asia Pac J Public Health* 2012, **24:**58-67.
31. Guo YLL, Lin YC, Sung FC, Huang SL, Ko YC, Lai JS, Su HJ, Shaw CK, Lin RS, Dockery DW: **Climate, traffic-related air pollutants, and asthma prevalence in middle-school children in Taiwan.** *Environ Health Perspect* 1999, **107:**1001-1006.
32. Sousa SIV, Ferraz C, Alvim-Ferraz MCM, Martins FG, Vaz LG, Pereira MC: **Spirometric tests to assess the prevalence of childhood asthma at Portuguese rural areas: Influence of exposure to high ozone levels.** *Environ Int* 2011, **37:**474-478.
33. Koshy G, Delpisheh A, Brabin BJ: **Trends in prevalence of childhood and parental asthma in Merseyside, 1991-2006.** *Journal of Public Health* 2010, **32:**488-495.
34. Hansen TE, Evjenth B, Holt J: **Increasing prevalence of asthma, allergic rhinoconjunctivitis and eczema among schoolchildren: three surveys during the period 1985-2008.** *Acta Paediatr* 2013, **102:**47-52.
35. Zollner IK, Weiland SK, Piechotowski I, Gabrio T, von Mutius E, Link B, Pfaff G, Kouros B, Wuthe J: **No increase in the prevalence of asthma, allergies, and atopic sensitisation among children in Germany: 1992-2001.** *Thorax* 2005, **60:**545-548.
36. Kim SY, Jung JY, Park MS, Kang YA, Kim EY, Kim SK, Chang J, Kim YS: **Increased Prevalence of Self-Reported Asthma Among**

Korean Adults: An Analysis of KNHANES I and IV Data. *Lung* 2013, **191:**281-288.
37. Bjerg A, Sandstrom T, Lundback B, Ronmark E: **Time trends in asthma and wheeze in Swedish children 1996-2006: prevalence and risk factors by sex.** *Allergy* 2010, **65:**48-55.
38. Bai J, Zhao J, Shen K-L, Xiang L, Chen A-H, Huang S, Huang Y, Wang J-S, Ye R-W: **Current Trends of the Prevalence of Childhood Asthma in Three Chinese Cities : A Multicenter Epidemiological Survey.** *Biomed Environ Sci* 2010, **23:**453-457.
39. Peat JK, Haby M, Spijker J, Berry G, Woolcock AJ: **Prevalence of asthma in adults in Busselton, Western-Australia.** *Br Med J* 1992, **305:**1326-1329.
40. Oraka E, Elizabeth HJ, King ME, Callahan DB: **Asthma Prevalence among US Elderly by Age Groups: Age Still Matters.** *J Asthma* 2012, **49:**593-599.
41. Barry AC, Mannino DM, Hopenhayn C, Bush H: **Exposure to Indoor Biomass Fuel Pollutants and Asthma Prevalence in Southeastern Kentucky: Results From the Burden of Lung Disease (BOLD) Study.** *J Asthma* 2010, **47:**735-741.
42. Kainu A, Pallasaho P, Piirila P, Lindqvist A, Sovijarvi A, Pietinalho A: **Increase in prevalence of physician-diagnosed asthma in Helsinki during the Finnish Asthma Programme: improved recognition of asthma in primary care? A cross-sectional cohort study.** *Prim Care Respir J* 2013, **22:**64-71.
43. Al Ghobain MO, Al-Hajjaj MS, Al Moamary MS: **Asthma prevalence among 16-to 18-year-old adolescents in Saudi Arabia using the ISAAC questionnaire.** *Bmc Public Health* 2012, **12**.
44. To T, Stanojevic S, Moores G, Gershon AS, Bateman ED, Cruz AA, Boulet L-P: **Global asthma prevalence in adults: findings from the cross-sectional world health survey.** *Bmc Public Health* 2012, **12**.
45. Wehrmeister FC, de Anselmo Peres KG: **Regional inequalities in the prevalence of asthma diagnosis in children: an analysis of the Brazilian National Household Sample Survey, 2003.** *Cad Saude Publica* 2010, **26:**1839-1852.
46. Strachan DP, Anderson HR, Limb ES, Oneill A, Wells N: **A national survey of asthma prevalence, severity, and treatment in Great-Britain.** *Arch Dis Child* 1994, **70:**174-178.
47. Bennoor KS, Hassan MR, Hossain MA, Mahmud AM, Ahmad MM, Habib GM: **Trends of asthma in Bangladesh: findings of the national asthma prevalence study -1999 and 2010.** *Respirology* 2011, **16:**212-212.
48. Andrea Rodriguez L, Jose Rey J, Berena Herrera A, Castro H, Niederbacher J, Maria Vera L, Libia Cala L, Bolivar F: **Respiratory symptoms associated with asthma prevalence and air pollution in preschool children.** *Biomedica* 2010, **30:**15-22.
49. Goh DYT, Chew FT, Quek SC, Lee BW: **Prevalence and severity of asthma, rhinitis, and eczema in Singapore schoolchildren.** *Arch Dis Child* 1996, **74:**131-135.

50. Shakurnia AH, Assar S, Afra M, Latifi M: **Prevalence of asthma among schoolchildren in Ahvaz, Islamic Republic of Iran.** *East Mediterr Health J* 2010, **16:**651-656.
51. Fedortsiv O, Brozek GM, Luchyshyn N, Kubey I, Lawson JA, Rennie DC, Zejda JE: **Prevalence of childhood asthma, rhinitis, and eczema in the Ternopil region of Ukraine - results of BUPAS study.** *Adv Med Sci* 2012, **57:**282-289.
52. Vonmutius E, Fritzsch C, Weiland SK, Roll G, Magnussen H: **Prevalence of asthma and allergic disorders among children in United Germany - a descriptive comparison.** *Br Med J* 1992, **305:**1395-1399.
53. Litonjua AA, Carey VJ, Weiss ST, Gold DR: **Race, socioeconomic factors, and area of residence are associated with asthma prevalence.** *Pediatr Pulmonol* 1999, **28:**394-401.
54. Rodriguez MA, Winkleby MA, Ahn D, Sundquist J, Kraemer HC: **Identification of population subgroups of children and adolescents with high asthma prevalence - Findings from the Third National Health and Nutrition Examination Survey.** *Arch Pediatr Adolesc Med* 2002, **156:**269-275.
55. Nriagu J, Martin J, Smith P, Socier D: **Residential hazards, high asthma prevalence and multimorbidity among children in Saginaw, Michigan.** *Sci Total Environ* 2012, **416:**53-61.
56. Haby MM, Peat JK, Marks GB, Woolcock AJ, Leeder SR: **Asthma in preschool children: prevalence and risk factors.** *Thorax* 2001, **56:**589-595.
57. Downs SH, Marks GB, Sporik R, Belosouva EG, Car NG, Peat JK: **Continued increase in the prevalence of asthma and atopy.** *Arch Dis Child* 2001, **84:**20-23.
58. Lukrafka JL, Fuchs SC, Moreira LB, Picon RV, Fischer GB, Fuchs FD: **Performance of the ISAAC Questionnaire to Establish the Prevalence of Asthma in Adolescents: A Population-Based Study.** *J Asthma* 2010, **47:**166-169.
59. Miller JE: **The effects of race/ethnicity and income on early childhood asthma prevalence and health care use.** *Am J Public Health* 2000, **90:**428-430.
60. Zobeiri M: **Prevalence, risk factors and severity of asthma symptoms in children of Kermanshah, IRAN: ISAAC phase I, II.** *Acta Med Iran* 2011, **49:**184-188.
61. Yeh K-W, Ou L-S, Yao T-C, Chen L-C, Lee W-I, Huang J-L, Grp PS: **Prevalence and risk factors for early presentation of asthma among preschool children in Taiwan.** *Asian Pac J Allergy Immunol* 2011, **29:**120-126.
62. Joseph CLM, Foxman B, Leickly FE, Peterson E, Ownby D: **Prevalence of possible undiagnosed asthma and associated morbidity among urban schoolchildren.** *J Pediatr* 1996, **129:**735-742.
63. Kwon J-W, Kim B-J, Song Y, Seo J-H, Kim T-H, Yu J, Kim HB, Lee SY, Kim W-K, Kim K-W, et al: **Changes in the Prevalence of Childhood Asthma in Seoul from 1995 to 2008 and Its Risk Factors.** *Allergy Asthma Immunol Res* 2011, **3:**27-33.

64. Gergen PJ, Mullally DI, Evans R: **National survey of prevalence of asthma among children in United-States, 1976 to 1980.** *Pediatrics* 1988, **81:**1-7.
65. Jie Y, Isa ZM, Jie X, Ismail NH: **Asthma and Asthma-Related Symptoms among Adults of an Acid Rain-Plagued City in Southwest China: Prevalence and Risk Factors.** *Pol J Environ Stud* 2013, **22:**717-726.
66. Rerksuppaphol S, Rerksuppaphol L: **Carotenoid intake and asthma prevalence in Thai children.** *Pediatr Rep* 2012, **4:**e12-e12.
67. Fukutomi Y, Taniguchi M, Watanabe J, Nakamura H, Komase Y, Ohta K, Akasawa A, Nakagawa T, Miyamoto T, Akiyama K: **Time trend in the prevalence of adult asthma in Japan: findings from population-based surveys in Fujieda City in 1985, 1999, and 2006.** *Allergol Int* 2011, **60:**443-448.
68. de Marco R, Pesce G, Marcon A, Accordini S, Antonicelli L, Bugiani M, Casali L, Ferrari M, Nicolini G, Panico MG, et al: **The Coexistence of Asthma and Chronic Obstructive Pulmonary Disease (COPD): Prevalence and Risk Factors in Young, Middle-aged and Elderly People from the General Population.** *Plos One* 2013, **8**.
69. Bryant-Stephens T, West C, Dirl C, Banks T, Briggs V, Rosenthal M: **Asthma Prevalence in Philadelphia: Description of Two Community-Based Methodologies to Assess Asthma Prevalence in an Inner-City Population.** *J Asthma* 2012, **49:**581-585.
70. Hong S, Son DK, Lim WR, Kim SH, Kim H, Yum HY, Kwon H: **The prevalence of atopic dermatitis, asthma, and allergic rhinitis and the comorbidity of allergic diseases in children.** *Environ Health Toxicol* 2012, **27:**e2012006-e2012006.
71. Gudelj I, Mrkic Kobal I, Munivrana Skvorc H, Mise K, Vrbica Z, Plavec D, Tudoric N: **Intraregional differences in asthma prevalence and risk factors for asthma among adolescents in Split-Dalmatia County, Croatia.** *Med Sci Monit* 2012, **18:**PH43-50.
72. Waite DA, Eyles EF, Tonkin SL, Odonnell TV: **Asthma prevalence in Tokelauan children in 2 environments.** *Clin Allergy* 1980, **10:**71-75.
73. de Sousa JC, Santo ME, Colaco T, Almada-Lobo F, Yaphe J: **Asthma in an Urban Population in Portugal: A prevalence study.** *Bmc Public Health* 2011, **11**.
74. Zivkovic Z, Vukasinovic Z, Cerovic S, Radulovic S, Zivanovic S: **Prevalence of childhood asthma and allergies in Serbia and Montenegro.** *World J Pediatr* 2010, **6:**331-336.
75. Anthracopoulos MB, Pandiora A, Fouzas S, Panagiotopoulou E, Liolios E, Priftis KN: **Sex-specific trends in prevalence of childhood asthma over 30 years in Patras, Greece.** *Acta Paediatr* 2011, **100:**1000-1005.
76. Anthracopoulos MB, Fouzas S, Pandiora A, Panagiotopoulou E, Liolios E, Priftis KN: **Prevalence trends of rhinoconjunctivitis, eczema, and atopic asthma in Greek schoolchildren: four surveys during 1991-2008.** *Allergy Asthma Proc* 2011, **32:**56-62.
77. Burr ML, Butland BK, King S, Vaughan-Williams E: **Changes in asthma prevalence: two surveys 15 years apart.** *Arch Dis Child* 1989, **64:**1452-1456.

78. Weitzman M, Gortmaker SL, Sobol AM, Perrin JM: **Recent trends in the prevalence and severity of childhood asthma.** *JAMA* 1992, **268:**2673-2677.
79. Chittleborough CR, Taylor AW, Dal Grande E, Gill TK, Grant JF, Adams RJ, Wilson DH, Ruffin RE, Team NWAHS: **Gender differences in asthma prevalence: Variations with socioeconomic disadvantage.** *Respirology* 2010, **15:**107-114.
80. Ekici A, Ekici M, Kocyigit P, Karlidag A: **Prevalence of Self-Reported Asthma in Urban and Rural Areas of Turkey.** *J Asthma* 2012, **49:**522-526.
81. Broms K, Norback D, Eriksson M, Sundelin C, Svardsudd K: **Prevalence and co-occurrence of parentally reported possible asthma and allergic manifestations in pre-school children.** *Bmc Public Health* 2013, **13**.
82. Clifford RD, Radford M, Howell JB, Holgate ST: **Prevalence of respiratory symptoms among 7-year-old and 11-year-old schoolchildren and association with asthma.** *Arch Dis Child* 1989, **64:**1118-1125.
83. Arif AA, Delclos GL, Lee ES, Tortolero SR, Whitehead LW: **Prevalence and risk factors of asthma and wheezing among US adults: an analysis of the NHANES III data.** *Eur Respir J* 2003, **21:**827-833.
84. Oraka E, King ME, Callahan DB: **Asthma and Serious Psychological Distress Prevalence and Risk Factors Among US Adults, 2001-2007.** *Chest* 2010, **137:**609-616.
85. Ma J, Xiao L, Knowles SB: **Obesity, insulin resistance and the prevalence of atopy and asthma in US adults.** *Allergy* 2010, **65:**1455-1463.
86. Akinbami LJ, Lynch CD, Parker JD, Woodruff TJ: **The association between childhood asthma prevalence and monitored air pollutants in metropolitan areas, United States, 2001-2004.** *Environ Res* 2010, **110:**294-301.
87. Akinbami LJ, Moorman JE, Liu X: **Asthma prevalence, health care use, and mortality: United States, 2005-2009.** *National health statistics reports* 2011**:**1-14.
88. Akinbami LJ, Moorman JE, Bailey C, Zahran HS, King M, Johnson CA, Liu X: **Trends in asthma prevalence, health care use, and mortality in the United States, 2001-2010.** *NCHS data brief* 2012**:**1-8.
89. Zahran HS, Bailey C, Garbe P: **Vital Signs: Asthma Prevalence, Disease Characteristics, and Self-Management Education-United States, 2001-2009 (Reprinted from MMWR, vol 60, pg 547-552, 2011).** *JAMA* 2011, **305:**2514-2516.
90. Moorman JE, Zahran H, Truman BI, Molla MT, Centers for Disease C, Prevention: **Current asthma prevalence - United States, 2006-2008.** *Morbidity and mortality weekly report Surveillance summaries (Washington, DC : 2002)* 2011, **60 Suppl:**84-86.
91. Frazier JC, Loveland KM, Zimmerman HJ, Helgerson SD, Harwell TS: **Prevalence of Asthma Among Adults in Metropolitan Versus Nonmetropolitan Areas in Montana, 2008.** *Prev Chronic Dis* 2012, **9**.

92. Coker TR, Kaplan RM, Chung PJ: **The Association of Health Insurance and Disease Impairment with Reported Asthma Prevalence in U.S. Children.** *Health Serv Res* 2012, **47:**431-445.
93. Musafiri S, van Meerbeeck J, Musango L, Brusselle G, Joos G, Seminega B, Rutayisire C: **Prevalence of atopy, asthma and COPD in an urban and a rural area of an African country.** *Respir Med* 2011, **105:**1596-1605.
94. Rodriguez A, Vaca M, Oviedo G, Erazo S, Chico ME, Teles C, Barreto ML, Rodrigues LC, Cooper PJ: **Urbanisation is associated with prevalence of childhood asthma in diverse, small rural communities in Ecuador.** *Thorax* 2011, **66:**1043-1050.
95. Duggan EM, Sturley J, Fitzgerald AP, Perry IJ, Hourihane JOB: **The 2002-2007 trends of prevalence of asthma, allergic rhinitis and eczema in Irish schoolchildren.** *Pediatr Allergy Immunol* 2012, **23:**464-471.
96. Anderson HR, Butland BK, Strachan DP: **Trends in prevalence and severity of childhood asthma.** *Br Med J* 1994, **308:**1600-1604.
97. Jain A, Bhat HV, Acharya D: **Prevalence of Bronchial Asthma in Rural Indian Children: A Cross Sectional Study from South India.** *Indian J Pediatr* 2010, **77:**31-35.
98. Sanchez-Lerma B: **High Prevalence of Asthma and Allergic Diseases in Children Aged 6 and 7 Years From the Canary Islands: The International Study of Asthma and Allergies in Childhood (vol 19, pg 383, 2009).** *J Investig Allergol Clin Immunol* 2012, **22:**79-79.
99. Luna MdFGd, Almeida PCd, Silva MGCd: **Asthma and rhinitis prevalence and co-morbidity in 13-14-year-old schoolchildren in the city of Fortaleza, Ceara State, Brazil.** *Cad Saude Publica* 2011, **27:**103-112.
100. de Luna MdFG, Fischer GB, de Luna JRG, da Silva MGC, de Almeida PC, Chiesa D: **Prevalences of asthma and rhinitis among adolescents in the city of Fortaleza, Brazil: temporal changes.** *J Bras Pneumol* 2013, **39:**128-137.
101. Matos Povoas Juca SCB, Takano OA, Lacerda Moraes LS, Guimaraes LV: **Asthma prevalence and risk factors in adolescents 13 to 14 years of age in Cuiaba, Mato Grosso State, Brazil.** *Cad Saude Publica* 2012, **28:**689-697.
102. Robinson CL, Baumann LM, Gilman RH, Romero K, Manuel Combe J, Cabrera L, Hansel NN, Barnes K, Gonzalvez G, Wise RA, et al: **The Peru Urban versus Rural Asthma (PURA) Study: methods and baseline quality control data from a cross-sectional investigation into the prevalence, severity, genetics, immunology and environmental factors affecting asthma in adolescence in Peru.** *Bmj Open* 2012, **2**.
103. Delmas MC, Guignon N, Leynaert B, Annesi-Maesano I, Com-Ruelle L, Gonzalez L, Fuhrman C: **Prevalence and control of asthma in young children in France.** *Rev Mal Respir* 2012, **29:**688-696.
104. Burney P, Chinn S, Luczynska C, Jarvis D, Vermeire P, Bousquet J, Nowak D, Prichard J, deMarco R, Rijcken B, et al: **Variations in the prevalence of respiratory symptoms, self-reported asthma**

attacks, and use of asthma medication in the European community respiratory health survey (ECRHS). *Eur Respir J* 1996, **9:**687-695.
105. Julia-Serda G, Cabrera-Navarro P, Acosta-Fernandez O, Martin-Perez P, Losada-Cabrera P, Garcia-Bello MA, Carrillo-Diaz T, Anto-Boque J: **High prevalence of asthma and atopy in the Canary Islands, Spain.** *Int J Tuberc Lung Dis* 2011, **15:**536-541.
106. Mahboub BH, Al-Hammadi S, Rafique M, Sulaiman N, Pawankar R, Al Redha AI, Mehta AC: **Population prevalence of asthma and its determinants based on European Community Respiratory Health Survey in the United Arab Emirates.** *Bmc Pulmonary Medicine* 2012, **12**.
107. Alves GdC, Santos DN, Feitosa CA, Barreto ML: **Community violence and childhood asthma prevalence in peripheral neighborhoods in Salvador, Bahia State, Brazil.** *Cad Saude Publica* 2012, **28:**86-94.
108. Akinbami LJ, Schoendorf KC: **Trends in childhood asthma: Prevalence, health care utilization, and mortality.** *Pediatrics* 2002, **110:**315-322.
109. Agrawal S: **Effect of Indoor Air Pollution from Biomass and Solid Fuel Combustion on Prevalence of Self-Reported Asthma among Adult Men and Women in India: Findings from a Nationwide Large-Scale Cross-Sectional Survey.** *J Asthma* 2012, **49:**355-365.
110. Agrawal S, Pearce N, Ebrahim S: **Prevalence and risk factors for self-reported asthma in an adult Indian population: a cross-sectional survey.** *Int J Tuberc Lung Dis* 2013, **17:**275-282.
111. Burney PGJ, Chinn S, Rona RJ: **Has the prevalence of asthma increased in children - evidence from the national study of health and growth 1973-86.** *Br Med J* 1990, **300:**1306-1310.
112. Krstic G: **Asthma Prevalence Associated with Geographical Latitude and Regional Insolation in the United States of America and Australia.** *Plos One* 2011, **6**.
113. Sousa CAd, Cesar CLG, Barros MBdA, Carandina L, Goldbaum M, Pereira JCR: **Prevalence of asthma and risk factors associated: population based study in Sao Paulo, Southeastern Brazil, 2008-2009.** *Rev Saude Publica* 2012, **46:**825-833.
114. Moreira SM, Ribeiro ACG, Fonseca M, Lourenco O, Taborda-Barata L: **Comparative study of the prevalence, clinical features, sensitisation profiles and risk factors for Bronchial Asthma between elderly and young adults in Cova da Beira Portugal.** *Eur J Med Res* 2010, **15:**216-216.
115. Parasuramalu BG, Huliraj N, Rudraprasad BM, Prashanth Kumar SP, Ramesh Masthi NR, Gangaboraiah: **Prevalence of bronchial asthma and its association with smoking habits among adult population in rural area.** *Indian J Public Health* 2010, **54:**165-168.
116. Woolcock AJ, Peat JK, Salome CM, Yan K, Anderson SD, Schoeffel RE, McCowage G, Killalea T: **Prevalence of bronchial hyperresponsiveness and asthma in a rural adult-population** *Thorax* 1987, **42:**361-368.

117. Cornell AG, Mellins RB, Acosta LM, Rundle AG, Miller RL, Kinney PL, Yan B, Perera FP, Jacobson JS, Goldstein IF, et al: **Exhaled No Is Elevated With Exposure To Black Carbon And Dust Mite Allergen Among 7 And 8-Year-Old Children Living In High And Low Asthma Prevalence Neighborhoods In New York City.** *Am J Respir Crit Care Med* 2011, **183**.
