# Supplementary material for: Operational definitions of asthma in recent epidemiological studies are inconsistent
Source: Clin Transl Allergy. 2014 Aug 4;4:24. doi: 10.1186/2045-7022-4-24 (PMC4136946; doi:10.1186/2045-7022-4-24)
Supplement: Additional file 3 — Definitions in the ten most cited and the ten most recent papers from the 117 included by Average citations per year at the end of 2013. [file 2045-7022-4-24-S3.pdf]

Additional File 3: Definitions in the ten most cited and the ten most recent papers from the 117 included by Average citations per year at the end of 2013.

First Author	Year	Journal	Times cited	Average citations per year	Current Asthma	Lifetime Asthma	Diagnosed Asthma
**Ten most cited papers**							
Pearce, N.	2007	Thorax	291	41.57		Ever had asthma	
Asher, M. I.	1998	European Respiratory Journal	644	40.25		Ever had asthma	
Akinbami, L. J.	2002	Pediatrics	408	34.00	Asthma in the last 12 months		
Burney, P.	1996	European Respiratory Journal	514	28.56	Asthma attack or currently taking asthma medication		
Burr, M. L.	1989	Archives Of Disease In Childhood	644	25.76	Ever had asthma and wheeze in the last 12 months		
Burney, P. G. J.	1990	British Medical Journal	482	20.08	Asthma in the last 12 months		
Moorman, J. E.	2011	Morbidity And Mortality Weekly Report. Surveillance Summaries	59	19.67	Diagnosed with asthma by a physician and still have asthma		
Vonmutius, E.	1992	British Medical Journal	331	15.05			Ever diagnosed with asthma by a physician or health professional
Gergen, P. J.	1988	Pediatrics	383	14.73			Diagnosed with asthma by a physician or wheezing in the last 12 months, apart from cold or the flu
Robertson, C. F.	1991	British Medical Journal	313	13.61		Ever had asthma	
**Ten most recent papers**							
Hansen, T. E.	2013	Acta Paediatrica	2	2		Ever had asthma and/or one of the following: wheeze, cough or acute shortness of breath due to external factors	
de Marco, R.	2013	Plos One	1	1			Ever had asthma and medical confirmation
Kim, S. Y.	2013	Lung	1	1			Ever diagnosed with asthma by a physician or health professional
Kainu, A.	2013	Primary Care Respiratory Journal	1	1			Ever diagnosed with asthma by a physician or health professional
Agrawal, S.	2013	International Journal Of Tuberculosis And Lung Disease	0	0	Asthma in the last 12 months		
Broms, K.	2013	BMC Public Health	0	0	At least one of the following: 1) Ever had asthma and wheezing in the last 12 months or 2) wheezing 4 times or more during the last 12 months or 3) physician diagnosis and wheezing in the last 12 months or 4) current use of inhalation steroids		
de Luna, M. d. F. G.	2013	Jornal Brasileiro De Pneumologia	0	0	Wheeze in the last 12 months		
Jie, Y.	2013	Polish Journal Of Environmental Studies	0	0			Diagnosed with asthma by a physician in the last 12 months
To, T.	2012	BMC Public Health	22	11			Ever diagnosed with asthma by a physician or health professional
Sanchez-Lerma, B.	2012	Journal Of Investigational Allergology And Clinical Immunology	8	4	Wheeze in the last 12 months		
