# Supplementary figures and images for: Operational definitions of asthma in recent epidemiological studies are inconsistent
Source: Clin Transl Allergy. 2014 Aug 4;4:24. doi: 10.1186/2045-7022-4-24 (PMC4136946; doi:10.1186/2045-7022-4-24)

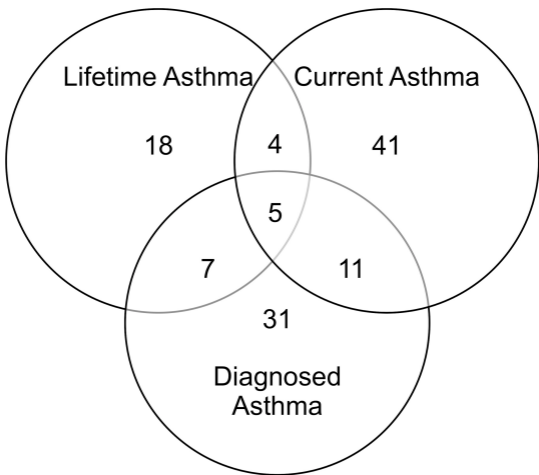

Supplement: Additional file 4 — Studies presenting three different subgroups of the definition of asthma. Of the 117 papers included, 34 provided a definition for lifetime asthma, 54 for diagnosed asthma and 61 for current asthma; 5 papers defined the three types of asthma. [file 2045-7022-4-24-S4.pdf]
