# Supplementary material for: Operational definitions of asthma in recent epidemiological studies are inconsistent
Source: Clin Transl Allergy. 2014 Aug 4;4:24. doi: 10.1186/2045-7022-4-24 (PMC4136946; doi:10.1186/2045-7022-4-24)
Supplement: Additional file 5 — Summary of studies with Current asthma definitions that used the International Study of Asthma and Allergies in Childhood (ISAAC) questionnaire (n = 18). [file 2045-7022-4-24-S5.pdf]

Additional File 5: Summary of studies with Current asthma definitions that used the International Study of Asthma and Allergies in Childhood (ISAAC) questionnaire (n=18).

Paper	Aim of the study	Study Setting	Methodology	Definition of current asthma
**Alves GdC, 2012[1]**	To determine the association between community violence and asthma symptoms.	Population-based sample of children Salvador-Baia, Brazil	Brazilian version of ISAAC questionnaire	Wheeze in the last 12 months and at least one of the following: any lifetime episode of asthma; wheezing during physical exercise; four or more episodes of wheezing in the previous year; or waking at night at least once a week due to wheezing in the previous year.
**Broms K, 2013[2]**	To make an in-depth analysis of the co-occurrence of possible asthma, allergic rhinitis, eczema, and food allergy.	Pre-school based sample of children from Sweden.	ISAAC questionnaire	One of the following 1) Ever had asthma and wheezing in the last 12 months or 2) wheezing 4 times or more during the last 12 months or 3) physician diagnosis and wheezing in the last 12 months or 4) current use of inhalation steroids
**de Farias MRdC, 2010[3]**	To assess the prevalence of symptoms of asthma	School-based sample of children and adolescents from Alta Floresta, Brazil.	Brazilian version of ISAAC I questionnaire	Wheeze in the last 12 months
**Dell SD, 2010[4]**	To assess the prevalence of asthma and allergic disease	School-based sample of children from Toronto - Ontario, Canada	ISAAC I protocol	Ever had asthma and was this confirmed by a doctor and wheeze and/or medication(s) for asthma or wheezing in the past 12 months
**Delmas MC, 2012[5]**	To estimate the prevalence of asthma	Data from 3 French national studies were analyzed: the Decennial Health Interview Survey conducted in 2003 among a representative sample of households and 2 surveys conducted among representative samples of schoolchildren from 9th grade in 2003–2004 and 5th grade in 2004–2005.	ISAAC questionnaire	Current wheeze and 1) Ever had an asthma attack or 2) taking medication(s) for asthma or wheezing
**Duggan EM, 2012[6]**	To compare the prevalence of asthma, allergic rhinitis and eczema in two cross-sectional studies performed 5 yr apart (2002 and 2007)	School-based sample of children from Cork City, Ireland	ISAAC questionnaire	Wheeze in the last 12 months
**Fiori NS, 2012[7]**	To compare asthma prevalence in two cross-sectional studies with a	Population-based sample of adults from	Brazilian version of ISAAC questions (and also included	Self-reported asthma in the last 12 months

	ten-year interval	Pelotas - Rio Grande do Sul, Brazil.	questions from ECRHS II)	
**Gomes de Luna MdF, 2013[8]**	To describe the prevalences of asthma and rhinitis, comparing the results with those obtained in a prevalence survey conducted in 2006-2007.	School-based sample of adolescents from Fortaleza - Ceará, Brasil	ISAAC I protocol	Wheeze in the last 12 months
**Gomes de Luna MdF, 2011[9]**	To assess the prevalence of asthma and rhinitis and the association between the two conditions.	School-based sample of adolescents from Fortaleza - Ceará, Brasil	ISAAC I protocol	Wheeze in the last 12 months
**Gudelj I, 2012[10]**	To compare intraregional prevalence of asthma	School base sample of adolescents from Split-Dalmatia County, Croatia	ISAAC questions (and also included questions from ECRHS II)	Wheeze in the last 12 months
**Hong S, 2012[11]**	To assess the prevalence of childhood atopic dermatitis, asthma, and allergic rhinitis	School-based sample of children from Seoul, Korea	ISAAC III questionnaire	Wheeze in the last 12 months
**Jain A, 2010[12]**	To assesse the prevalence of bronchial asthma and its association with socio-demographic factors	Community based sample study among children aged 6-15 yr in the rural field practice area of the department of Community Medicine at KMC, Manipal.	Kannada version of ISAAC questionnaire	Wheeze in the last 12 months
**Kwon J-W, 2011[13]**	To assess the prevalence of asthma and determine its risk factors	School-base sample of children from Seoul, Japan	Modified ISAAC questionnaire	Diagnosed with asthma in their lifetime and experienced wheezing in the last 12 months
**Matos Povoas Juca SCB, 2012[14]**	To estimate the prevalence of active asthma and identify associated risk factors.	School-based sample of adolescents from Cuiaba - Mato Grosso, Brazil.	ISAAC I protocol and modified brazilian version of ISAAC questionnaire (one question added)	Wheeze in the last 12 months
**Robinson CL, 2012[15]**	To compare the prevalence and severity of asthma in a urban and rural communities	Population-based sample of adolescents from Peru	Spanish version of ISAAC questionnaire	Wheeze and/or use of asthma medication in the last 12 months
**Rodriguez A, 2011[16]**	To explore the influence of urbanization in asthma prevalence	Population-based sample of children and adolescents from Esmeraldas, Ecuador	Questionnaire adapted from ISAAC II	Wheeze in the last 12 months
**Sanchez-Lerma B, 2012[17]**	To investigate the prevalence of bronchial asthma and allergic diseases	School-based sample of children from Canary Islands	ISAAC I protocol	Wheeze in the last 12 months
**Toledo MF, 2011[18]**	To assess the prevalence of asthma and allergies	School-based sample of adolescents from the city of Taubate, Sao Paulo, Brazil	ISAAC I questionnaire	Wheeze in the last 12 months

References

1. Alves GdC, Santos DN, Feitosa CA, Barreto ML: **Community violence and childhood asthma prevalence in peripheral neighborhoods in Salvador, Bahia State, Brazil.** *Cad Saude Publica* 2012, **28:**86-94.
2. Broms K, Norback D, Eriksson M, Sundelin C, Svardsudd K: **Prevalence and co-occurrence of parentally reported possible asthma and allergic manifestations in pre-school children.** *Bmc Public Health* 2013, **13**.
3. de Farias MRdC, Rosa AM, Hacon SdS, de Castro HA, Ignotti E: **Prevalence of asthma in schoolchildren in Alta Floresta- a municipality in the southeast of the Brazilian Amazon.** *Rev Bras Epidemiol* 2010, **13:**49-57.
4. Dell SD, Foty RG, Gilbert NL, Jerret M, To T, Walter SD, Stieb DM: **Asthma and allergic disease prevalence in a diverse sample of Toronto school children: results from the Toronto Child Health Evaluation Questionnaire (T-CHEQ) Study.** *Can Respir J* 2010, **17:**e1-6.
5. Delmas MC, Guignon N, Leynaert B, Annesi-Maesano I, Com-Ruelle L, Gonzalez L, Fuhrman C: **Prevalence and control of asthma in young children in France.** *Rev Mal Respir* 2012, **29:**688-696.
6. Duggan EM, Sturley J, Fitzgerald AP, Perry IJ, Hourihane JOB: **The 2002-2007 trends of prevalence of asthma, allergic rhinitis and eczema in Irish schoolchildren.** *Pediatr Allergy Immunol* 2012, **23:**464-471.
7. Fiori NS, Goncalves H, Dumith SC, Dropa Chrestani Cesar MA, Menezes AMB, Cardozo Macedo SE: **Ten-year trends in prevalence of asthma in adults in southern Brazil: comparison of two population-based studies.** *Cad Saude Publica* 2012, **28:**135-144.
8. de Luna MdFG, Fischer GB, de Luna JRG, da Silva MGC, de Almeida PC, Chiesa D: **Prevalences of asthma and rhinitis among adolescents in the city of Fortaleza, Brazil: temporal changes.** *J Bras Pneumol* 2013, **39:**128-137.
9. Luna MdFGd, Almeida PCd, Silva MGCd: **Asthma and rhinitis prevalence and co-morbidity in 13-14-year-old schoolchildren in the city of Fortaleza, Ceara State, Brazil.** *Cad Saude Publica* 2011, **27:**103-112.
10. Gudelj I, Mrkic Kobal I, Munivrana Skvorc H, Mise K, Vrbica Z, Plavec D, Tudoric N: **Intraregional differences in asthma prevalence and risk factors for asthma among adolescents in Split-Dalmatia County, Croatia.** *Med Sci Monit* 2012, **18:**PH43-50.
11. Hong S, Son DK, Lim WR, Kim SH, Kim H, Yum HY, Kwon H: **The prevalence of atopic dermatitis, asthma, and allergic rhinitis and the comorbidity of allergic diseases in children.** *Environ Health Toxicol* 2012, **27:**e2012006-e2012006.
12. Jain A, Bhat HV, Acharya D: **Prevalence of Bronchial Asthma in Rural Indian Children: A Cross Sectional Study from South India.** *Indian J Pediatr* 2010, **77:**31-35.
13. Kwon J-W, Kim B-J, Song Y, Seo J-H, Kim T-H, Yu J, Kim HB, Lee SY, Kim W-K, Kim K-W, et al: **Changes in the Prevalence of Childhood

Asthma in Seoul from 1995 to 2008 and Its Risk Factors. *Allergy Asthma Immunol Res* 2011, **3:**27-33.

14. Matos Povoas Juca SCB, Takano OA, Lacerda Moraes LS, Guimaraes LV: **Asthma prevalence and risk factors in adolescents 13 to 14 years of age in Cuiaba, Mato Grosso State, Brazil.** *Cad Saude Publica* 2012, **28:**689-697.
15. Robinson CL, Baumann LM, Gilman RH, Romero K, Manuel Combe J, Cabrera L, Hansel NN, Barnes K, Gonzalvez G, Wise RA, et al: **The Peru Urban versus Rural Asthma (PURA) Study: methods and baseline quality control data from a cross-sectional investigation into the prevalence, severity, genetics, immunology and environmental factors affecting asthma in adolescence in Peru.** *Bmj Open* 2012, **2**.
16. Rodriguez A, Vaca M, Oviedo G, Erazo S, Chico ME, Teles C, Barreto ML, Rodrigues LC, Cooper PJ: **Urbanisation is associated with prevalence of childhood asthma in diverse, small rural communities in Ecuador.** *Thorax* 2011, **66:**1043-1050.
17. Sanchez-Lerma B: **High Prevalence of Asthma and Allergic Diseases in Children Aged 6 and 7 Years From the Canary Islands: The International Study of Asthma and Allergies in Childhood (vol 19, pg 383, 2009).** *J Investig Allergol Clin Immunol* 2012, **22:**79-79.
18. Toledo MF, Rozov T, Leone C: **Prevalence of asthma and allergies in 13-to 14-year-old adolescents and the frequency of risk factors in carriers of current asthma in Taubate, Sao Paulo, Brazil.** *Allergol Immunopathol (Madr)* 2011, **39:**284-290.
